# The Glycine- and Proline-Rich Protein AtGPRP3 Negatively Regulates Plant Growth in *Arabidopsis*

**DOI:** 10.3390/ijms21176168

**Published:** 2020-08-26

**Authors:** Xiaojing Liu, Xin Wang, Xin Yan, Shaobo Li, Hui Peng

**Affiliations:** 1 Key Laboratory of Molecular Biology and Gene Engineering of Jiangxi Province, College of Life Science, Nanchang University, Nanchang 330031, China; 407311416073@email.ncu.edu.cn (X.L.); wangxin@ncu.edu.cn (X.W.); xinyan@ncu.edu.cn (X.Y.); 2 The Genome Center and Department of Plant Sciences, University of California, Davis, CA 95616, USA; 3 College of Life Sciences, Guangxi Normal University, Guilin 541004, China

**Keywords:** *Arabidopsis*, glycine- and proline-rich protein, protein interaction, seedling growth

## Abstract

Glycine- and proline-rich proteins (GPRPs) comprise a small conserved family that is widely distributed in the plant kingdom. GPRPs are relatively short peptides (<200 amino acids) that contain three typical domains, including an N-terminal XYPP-repeat domain, a middle hydrophobic domain rich in alanine, and a C-terminal HGK-repeat domain. These proteins have been proposed to play fundamental roles in plant growth and environmental adaptation, but their functions remain unknown. In this study, we selected an *Arabidopsis* GPRP (AtGPRP3) to profile the physiological role of GPRPs. Transcripts of *AtGPRP3* could be detected in the whole *Arabidopsis* plant, but greater amounts were found in the rosette, followed by the cauline. The AtGPRP3::GFP fusion protein was mainly localized in the nucleus. The overexpression and knockout of *AtGPRP3*, respectively, retarded and accelerated the growth of *Arabidopsis* seedlings, while the increase in the growth rate of *atgprp3* plants was offset by the complementary expression of *AtGPRP3*. CAT2 and CAT3, but not CAT1, interacted with AtGPRP3 in the nuclei of *Arabidopsis* protoplasts. The knockout of *CAT2* by CRISPR-Cas9 retarded the growth of the *Arabidopsis* seedlings. Together, our data suggest that AtGPRP3 negatively regulates plant growth, potentially through CAT2 and CAT3.

## 1. Introduction

In plants, glycine- and proline-rich proteins (GPRPs) were first characterized in *Arabidopsis* [1]. It is known that plant GPRPs usually have three conserved domains, an N-terminal XYPP domain, a central hydrophobic domain, and an HGK domain at the C terminus [1,2]. The XYPP motif was considered to form beta-turn helices [3] and interact with some cytoplasmic components [4] or intercellular proteins [2]. The central hydrophobic domain rich in alanine was characterized as a transmembrane domain [1,2]. The HGK-repeat domain at the C terminus was presumed to form a disordered coil and play important roles in molecular interactions [5,6]. However, the molecular effects of these conserved domains are still unclear in plants [2,7], and the biological functions of the genes coding for these plant GPRPs have not been well resolved [2].

To date, although the biological functions of plant *GPRP* genes and their molecular mechanisms have seldom been explored, the existing experimental data show that they should play important roles in plant growth and development, as well as adversity adaptation [1,2,8]. For example, in common plantain, *PmGPRPs* have been observed to be highly expressed in vascular tissues where transport is very active, such as the phloem and epidermis, suggesting the potential vesicular traffic or internal cellular transport roles of *PmGPRPs* [8]. The expression of GPRPs such as chickpea GPRP (*CarPRP1*) and soybean GPRPs (*GmGPRP1*, *3*, and *4*) are stimulated by external treatments, including drought, salt, cold, heat, bacterial (*Pseudomonas syringae*) infection, and phytohormones (methyl jasmonate and salicylic acid) [2,9,10]. A sweet potato GPRP (*IbPGAHRP1*) was found to enhance cold and drought resistance in yeast recombinants [11], and a Sorghum GPRP (*SbGPRP1*) improved tolerance towards a bacterial phytopathogen (*Rhodococcus fascians*) infection in tobacco [9]. However, the machinery behind these findings and the function of GPRPs in plant growth remain unknown.

Crop production is highly determined by plant growth, which is regulated by many factors including limiting nutrients, photobiology, plant hormone signaling, reactive oxygen species, and transcription factors [12,13,14,15]. Nitrogen, as a limiting nutrient for plant growth and crop yield, is a main component of fertilizers and heavily used in modern agriculture [14]. AP2 transcription factors play important roles in regulating plant growth and development [13]. Light is crucial for plant life, and the perception of the light environment dictates plant growth, morphology, and developmental changes [12].

Emerging evidence shows the important role of GPRPs in both plant development and defense response [1,2]. However, the function of the GPRP family is poorly understood. Thus, research is presently searching for the interacting factors of the stress-related glycine- and proline-rich protein (GPRP). To elucidate the molecular mechanism of the AtGPRP3 mediation of plant development, we previously generated an *Arabidopsis* cDNA library for a yeast two-hybrid analysis and identified 26 interactor candidates of AtGPRP3. To verify the accuracy of the interactions, we used AtGPRP3 as the bait and the candidates as the prey in a yeast two-hybrid analysis. Finally, we found that there is a certain interaction between AtGPRP3 and CATALASE2 (CAT2). In this study, we mainly report how AtGPRP3 affected the plant growth of 10-day *Arabidopsis* seedlings (without roots) by interacting with CAT2. Our results will be useful for further analyzing the biological functions of AtGPRP3 and other plant GPRPs, as well as their molecular mechanisms.

## 2. Results

### 2.1. AtGPRP3 Is a Member of a Small and Conserved Gene Family

**Figure 1:**
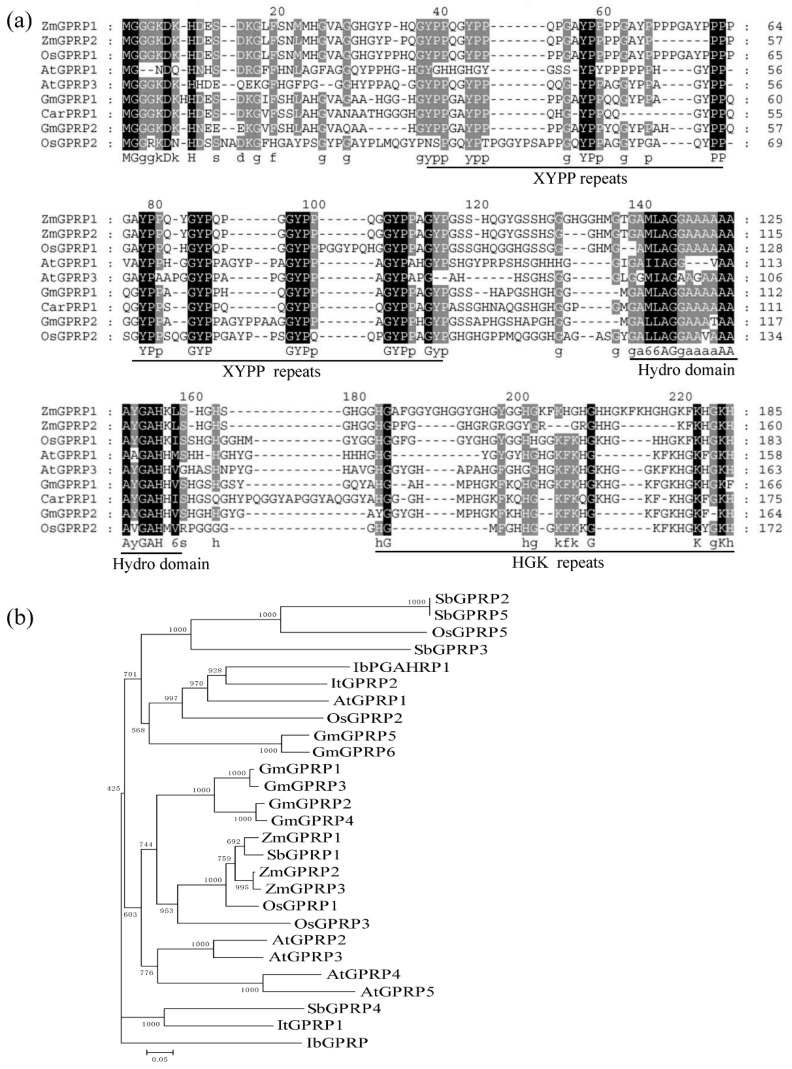
Multiple sequence alignment and phylogenetic analysis of glycine- and proline-rich proteins (GPRPs). (**a**) Multiple sequence alignment of GPRPs from different species. Identical amino acids are shaded in black, and similar amino acids are shaded in grey. (**b**) Phylogenetic tree of GPRPs from different species. Total of 22 GPRPs were identified from genomes of six species including *Arabidopsis thaliana*, *Oryza sativa*, *Zea mays*, *Glycine max*, *Sorghum bicolor*, *Ipomoea batatas*, and *Ipomoea trifida* used for the construction of the phylogenetic tree. Accession numbers of all GPRPs were presented in Table S1.

In our previous study, a chickpea GPRP gene (CarPRP1) identified from a drought-stress cDNA library was experimentally documented as a modulator in response to abiotic stresses, such as drought and high salinity [2,9,10]. To determine the physiological role of the GPRP family, AtGPRP3, the ortholog of CarPRP1 in Arabidopsis, was selected for further analyses. Running BLAST on the Arabidopsis genome database (https://www.arabidopsis.org/) revealed five GPRP-like genes (AtGPRP1-5). These genes encoded a group of short peptides (<200 amino acids) that showed a relatively high similarity (49–83%) to each other. The predicted protein of AtGPRP3 consisted of 179 amino acids and included 29.6% glycine, 13.4% proline, 12.8% histidine, and 12.8% alanine, presenting a typical residue preference. Comprehensive alignment of the GPRPs from various species, including Arabidopsis, rice, soybean, chickpea, and maize, revealed the sequence conservation in the plant kingdom. These conserved proteins shared three domains, including an XYPP-repeat domain at the N-terminal, an alanine (A)-enriched hydro domain in the middle, and a histidine (H)-glycine (G)-lysine (K)-repeat domain at the C-terminal (Figure 1a). In the polyphyletic tree generated with GPRPs from seven monocotyledon and dicotyledon species (Arabidopsis, soybean, rice, maize, sorghum, and sweet potato), paralog pairs such as AtGPRP2 and 3 appeared in most examined species (Figure 1b), indicating the duplication of a GPRP following the differentiation of families. On the other hand, some GPRP paralogs from species such as OsGPRP5 and 3 were classified into separate branches, while some orthologs from different species, such as OsGPRP5 and GmGPRP5, were grouped into the same branch, suggesting that the duplication of these GPPRs occurred prior to the differentiation of monocotyledon from dicotyledon.

### 2.2. AtGPRP3 Is Ubiquitously Expressed in Arabidopsis

**Figure 2:**
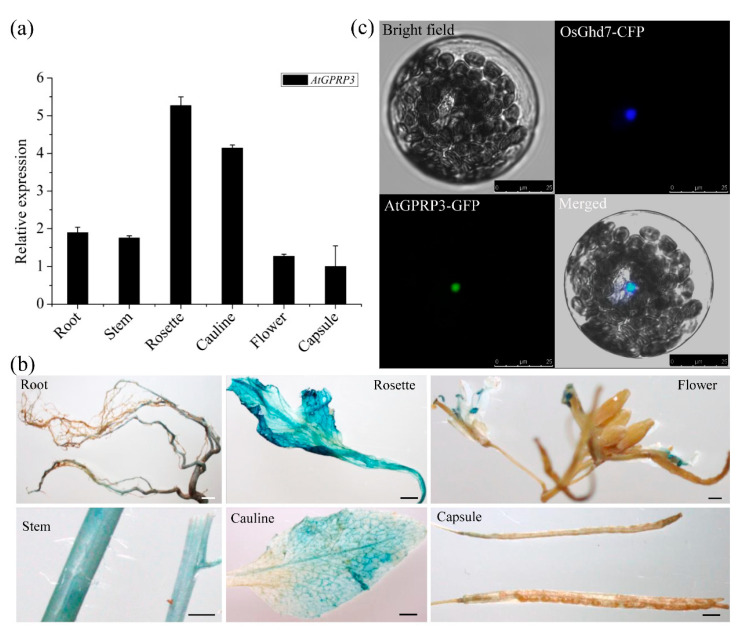
The expression and subcellular localization of AtGPRP3. (**a**) Tissue-specific expression of AtGPRP3. Total RNAs were extracted from different tissues including roots, stems, rosette, cauline, flowers, and capsules of *Col-0* plants grown under normal conditions. Relative expression was calculated with Cq values from qRT-PCR experiments. Data are mean ± SE for three replicates. (**b**) Tissue-specific distribution of AtGPRP3 transcripts. β-glucuronidase (GUS) staining was conducted with various tissues in transgenic plants carrying the pAtGPRP3::GUS reporter. Bar = 2 mm. (**c**) Subcellular localization of AtGPRP3. AtGPRP3-GFP and OsGhd7-CFP (a nuclear marker) were constitutively expressed in Arabidopsis protoplasts. The green fluorescent protein (GFP) and cyan fluorescence protein (CFP) fluorescence images were taken from the same cell and merged. Bar = 25 μm.

To identify the location in which the GPRP fulfills its functions, we first investigated the expression of AtGPRP3 in various tissues of wild-type Arabidopsis under normal growth conditions using quantitative RT-PCR (qRT-PCR). The results showed that AtGPRP3 was transcribed in all detected tissues, with a relatively high transcription level in rosette and cauline (Figure 2a). We then examined the spatial distribution of AtGPRP3 transcripts in wild-type plants under normal growth conditions with the GUS (β-glucuronidase) gene reporting system. A 2, 116 bp upstream sequence of the start codon ATG that represents the regulatory region of the AtGPRP3 gene was applied to drive the GUS reporter gene in the transformed plants. Consistent with the qRT-PCR results, strong expression of AtGPRP3 was observed in rosette and cauline, followed by stems and flowers. The lowest expression occurred in capsules. Notably, the transcripts of AtGPRP3 were not distributed in the whole flower, but there was substantial accumulation in the stamen and stigma (Figure 2b). Further, we assessed the subcellular localization of the AtGPRP3 protein in Arabidopsis protoplasts using the GFP reporter system. Transient expression of the fluorescent signal in the protoplast showed that the AtGPRP3-GFP was localized only in the nucleus. In transgenic tobacco, a GFP signal was detected in the nuclei of epidermal cells (Figure S1a). In addition, green fluorescent signals of the AtGPRP3-GFP were colocalized with Ghd7-CFP, a nuclear marker (Figure 2c), suggesting that AtGPRP3 is present in the nucleus.

### 2.3. Modification of AtGPRP3 Expression Affects Plant Growth

**Figure 3:**
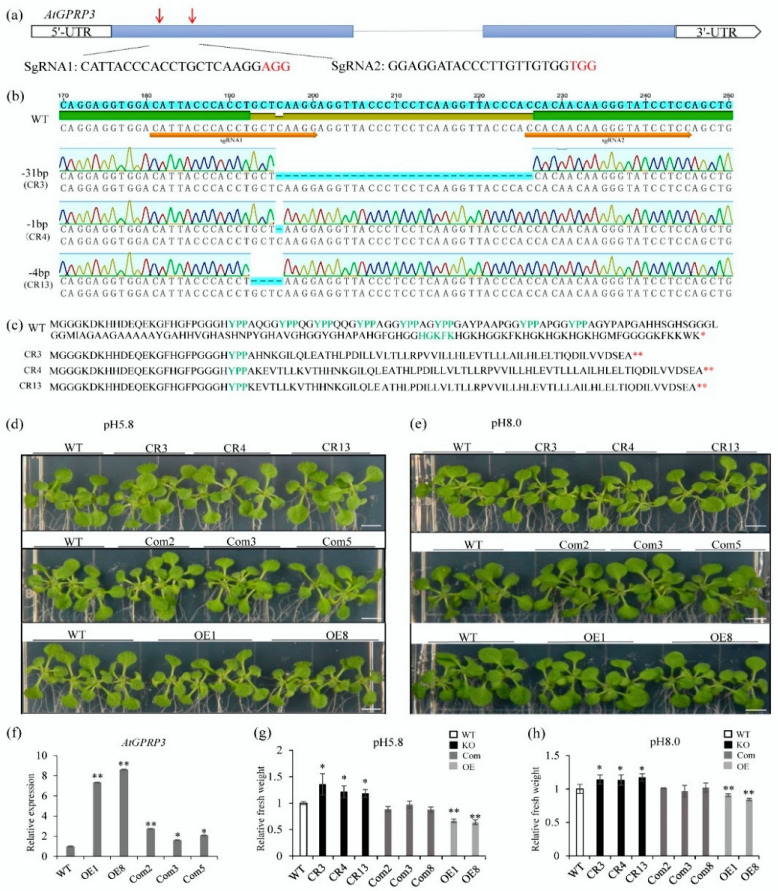
Creation of *atgprp3* knockout mutants by CRISPR/Cas9 and impact of modification of *AtGPRP3* expression on seedling growth. (**a**) CRISPR target sites of *AtGPRP3*. Untranslated regions (UTR), exons, and introns are labeled as open boxes, solid blue boxes, and lines, respectively. Target sites SgRNA1 and SgRNA2 were indicated with red arrows. (**b**) DNA sequences of CRISPR target area in *AtGPRP3* and *atgprp3* mutants. Target area sequences of three mutants (CR3, CR4, and CR13) are aligned to that of original *AtGPRP3* (wild-type, WT). Dashes indicate the missing nucleotides. (**c**) Polypeptide sequences of *AtGPRP3* (WT) and *atgprp3* mutants (CR3, CR4, and CR13). Stop codons are labeled with stars. (**d**) Phenotype of *Col-0* (WT), mutant (CR3, CR4, and CR13), complementation (Com2, Com3, and Com5), and overexpression (OE1 and OE8) line seedlings grown on MS medium at pH 5.8. (**e**) Phenotype of *Col-0*, mutant, complementation, and overexpression line seedlings grown on MS medium at pH 8.0. Bars = 6 mm. (**f**) Relative expression of *AtGPRP3* in leaves of *Col-0*, complementation, and overexpression line seedlings. Values are presented as means ± standard error (SE). Significance levels at *p* < 0.05 and *p* < 0.001 are labelled with single and double stars, respectively. (**g**) Relative fresh weight of *Col-0*, knockout (KO) mutant, complementation, and overexpression line seedlings on MS medium at pH 5.8. Values are presented as means ± standard error (SE). Significance levels at *p* < 0.05 and *p* < 0.01 are labelled with single and double stars, respectively. (**h**) Relative fresh weight of *Col-0*, knockout (KO) mutant, complementation, and overexpression line seedlings on MS medium at pH 8.0.

To reveal the physiological function of *AtGPRP3* in *Arabidopsis*, we first investigated the phenotype of the knockout mutant *atgprp3* generated by CRISPR-Cas9 technology. The two CRISPR targets were located in the first exon of *AtGPRP3* (Figure 3a and Table S1). Off-target detection towards the homologs of *AtGPRP3* showed no unexpected cleavage at either of the two CRISPR target sites (Table S2). Three transgenic lines (CR3, CR4, and CR13), up to the T_5_ generation, were selected for further analysis. DNA sequencing revealed that the genomes of CR3, CR4, and CR13 had −31 nt, −1 nt, and −4nt deletion, respectively, in the target region (Figure 3b). These deletions resulted in a shift of the open reading frames (ORF) that caused an abnormal termination of translation. The resulting peptides thus missed most of the XYPP repeats, the A-enriched hydro domain, and all HGK repeats (Figure 3c). Interestingly, all *atgprp3* seedlings did not show visible organ malformation but displayed an obviously larger size compared to the WT ones. The fresh shoot weights of the 11-day seedlings of CR3, CR4, and CR13 were, respectively, 23.0%, 19.7%, and 20.9% higher than those of the WT plants on the acidic (pH = 5.8) medium. A similar observation was also obtained for the seedlings growing on the basic (pH = 8.0) medium (Figure 3d–h). To further confirm the impact of the *AtGPRP3* expression level on seedling growth, we introduced *AtGPRP3* into WT and the *atgprp3* mutant plants to generate overexpression and complementary expression lines, respectively. Transcriptional analysis revealed that the *AtGPRP3* mRNA levels were higher in the overexpression and complementary lines than in the WT ones, although *AtGPRP3* mRNA’s abundance in the overexpression lines was approximately four times of that in the complementary lines (Figure 3f). The recouped expression of *AtGPRP3* in the knockout mutant plants led to a clear growth rate of the seedlings similar to that of WT on both the acidic and basic mediums, while the overexpression of *AtGPRP3* retarded seedling growth (Figure 3d,e,g,h).

### 2.4. AtGPRP3 Interacts with CAT2 and 3 But Not CAT1

**Figure 4:**
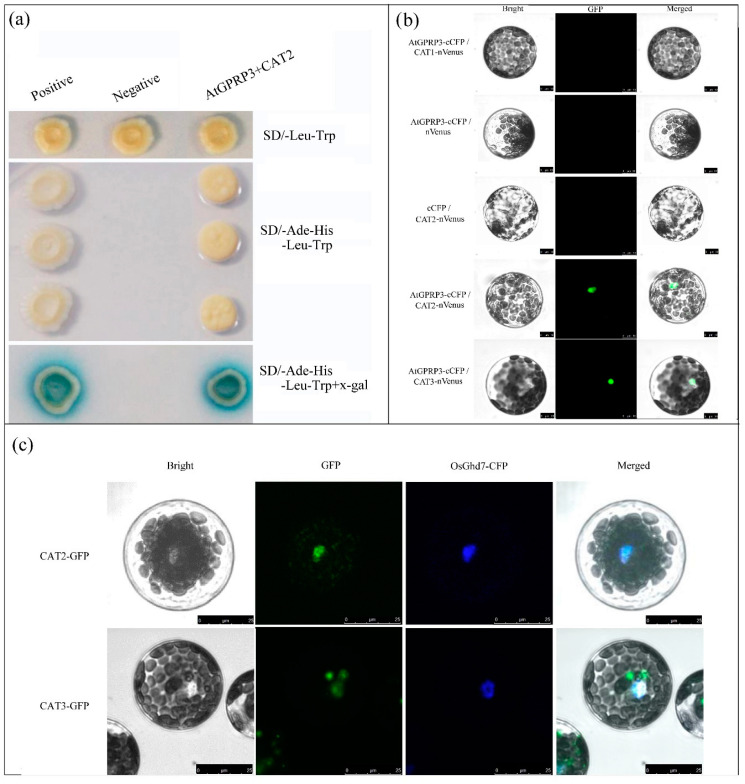
Interaction of AtGPRP3 with catalases and subcellular localization of CAT2 and CAT3. (**a**) Interactions of AtGPRP3 with CAT2 in yeast. Yeast transformants carrying plasmid BD53 and AD-T (positive control), empty vectors pGBKT7 and pGADT7 (negative control), and constructs of AtGPRP3 and CAT2 were cultured on selective mediums SD/-Leu/-Trp and SD/-Leu/-Trp/-His/-Ade. X-gal was used to detect the interaction of AtGPRP3 with CAT2. (**b**) Interactions of AtGPRP3 with three catalases in *Arabidopsis* protoplast. AtGPRP3 fused with the N-terminus of CFP was cotransformed into protoplasts, respectively, with CAT1, CAT2, and CAT3 fused with the C-terminus of CFP (cCFP) in bimolecular fluorescence complementation (BiFC) experiment. Bright, GFP, and Merged indicate bright field images, GFP fluorescence, and merged images, respectively. Bar = 10 μm. (**c**) Subcellular localization of CAT2 and AtGPRP3 in Arabidopsis protoplast. CAT2-GFP and OsGhd7-CFP (a nuclear marker) were constitutively expressed in Arabidopsis protoplast. The GFP and CFP fluorescence images were taken from the same cell and merged. Scale bar = 25 μm.

To elucidate the molecular mechanism of AtGPRP3′s mediation of plant development, we previously generated an *Arabidopsis* cDNA library for yeast two-hybrid analysis and identified 26 interactor candidates of AtGPRP3, including CAT2 (catalase 2, AT4G35090). CAT2 was selected for further study, as catalases have been reported to play an important role in both plant development and stress response. To verify the interactions of CAT2 and AtGPRP3, we selected AtGPRP3 and CAT2 as the bait and prey, respectively, in the yeast two-hybrid analysis. Finally, we found that there were, indeed, interactions between AtGPRP3 and CAT2. As shown in Figure 4a, yeast colonies coexpressing AtGPRP3 and CAT2 or BD53 and AD-T (positive control) were able to grow on the deficient medium lacking Ade and His and also displayed a blue color in the presence of X-gal, whereas colonies carrying empty vectors (negative control) did not grow on the deficient medium, indicating the physical interaction of AtGPRP3 with CAT2 in yeast cells.

To assess if AtGPRP3 also interacts with CAT2 in plant cells, a bimolecular fluorescence complementation (BiFC) test was performed with the *Arabidopsis* protoplasts. Fluorescence was observed in the nuclei of the protoplasts coexpressing AtGPRP3 and CAT2, indicating that the two proteins interact in the nucleus (Figure 4b, c and Figure S1b). Parallel experiments were conducted to examine if AtGPRP3 also interacts with other catalases (CAT1 and CAT3). Fluorescence was detected in the nuclei of cells coexpressing AtGPRP3 and CAT3 but not in the cells expressing CAT1, suggesting that AtGPRP3 does not interact with CAT1 but instead with CAT3 in the nucleus (Figure 4b).

To assess the distribution of the two catalases interacting with AtGPRP3 in cells, subcellular localization tests were performed on the Arabidopsis protoplasts. As shown in Figure 4c, fluorescence signals were observed in both the nuclei and cytosol in cells expressing CAT2-GFP or CAT3-GFP fusion proteins, suggesting that CAT2 and CAT3 exist in both cellular organelles.

### 2.5. Knockout of CAT2 Slows the Growth of Arabidopsis Seedlings

**Figure 5:**
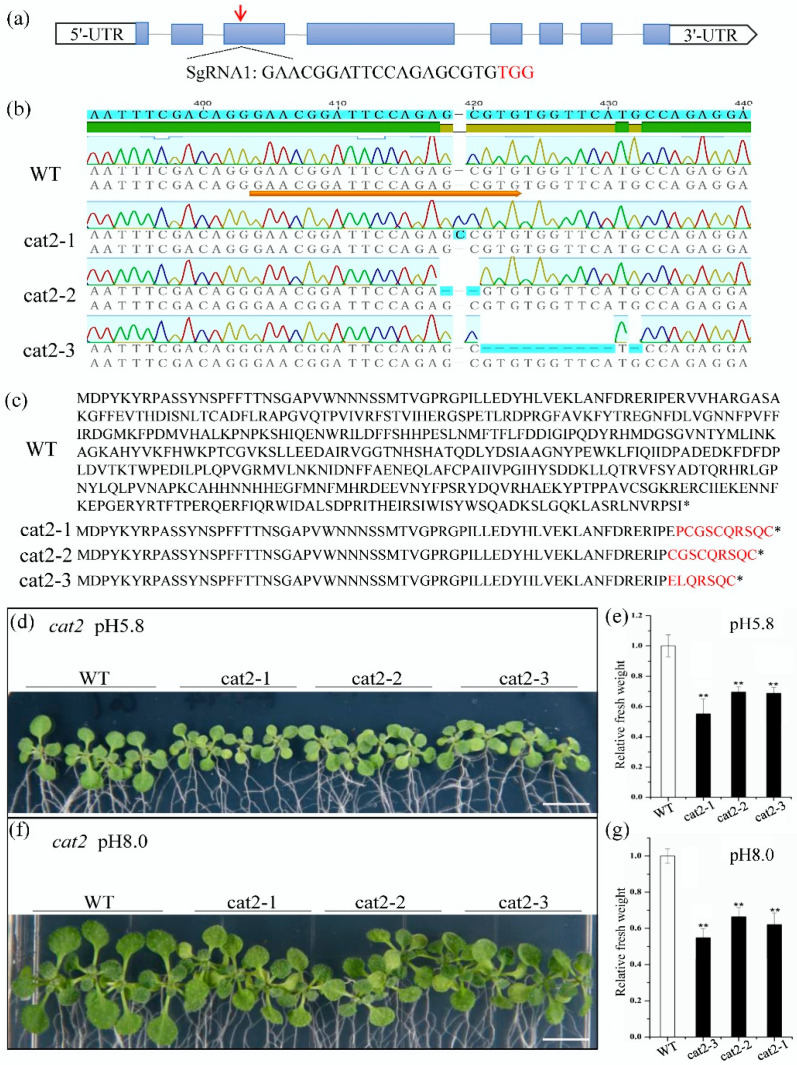
Phenotypic analysis of *CAT2*-CRISPR lines. (**a**) The target site disrupts the third exon of *CAT2*. The red arrows show target sites. (**b**) Genomic of *CAT2* from WT and mutants cat2-1, cat2-2, and cat2-3 generated using CRISPR-Cas9 technology. (**c**) Polypeptide sequences comparison of the WT and the mutants, showing that CRISPR-Cas9 technology lead to truncations of CAT2 protein. * Stop codon. (**d**,**f**) Phenotype of *Col-0*, CRISPR lines, and WT seedlings grown on MS medium under pH 5.8 and 8.0, respectively. (**e**,**g**) Fresh shoot weight of *Col-0* and CRISPR lines seedlings germinated and grown on MS medium under pH 5.8 and 8.0, respectively. Data are given as means ± SE of five biological replicates. * and ** represent significant difference at the 5% and 1% levels, respectively.

To examine the role of *CAT2* on plant growth, we investigated the phenotype of the knockout mutant *cat2* with CRISPR-Cas9 technology. The phenotypes of transformants overexpressing a catalase were not examined in this study because catalase genes are highly expressed in plants, and overexpression of a catalase normally does not increase catalase activity [16]. The CRISPR target was selected from the third exon of *CAT2* (Figure 5a). Off-target detection of the homologs (*CAT1* and *CAT3*) of *CAT2* did not find any cleavage at the CRISPR target site (Table S2). Three mutant lines (*cat2-1*, *cat2-2*, and *cat2-3*), up to the T_3_ generation, were selected for further analysis. DNA sequencing revealed that *cat2-1*, *cat2-2,* and *cat2-3* had a +1nt insertion, a −2nt deletion, and a −11nt deletion in the CRISPR target site, respectively (Figure 5b). These changes resulted in heavily truncated catalases that missed the catalytic site (Figure 5c). All *cat2* mutant seedlings exhibited smaller sizes than the WT ones after being cultured on both acidic (pH = 5.8) and basic (pH = 8.0) media for 11 days (Figure 5d,f). The fresh shoot weights of *cat2* mutant seedlings were also significantly lower than those of the WT ones (Figure 5e,g).

## 3. Discussion

GPRPs widely exist in plant species such as *Arabidopsis*, rice, maize, sorghum, tomato, tobacco, sweet potato, chickpea, and soybean (Figure 1a) [1,2,9,11,17,18]. These GPRPs comprise a small family that usually has less than six members in a species (Figure 1b) [1,2]. These short peptides (<200 amino acids) show high similarity (49–83%) to each other and contain three typical domains, including an N-terminal XYPP-repeat domain, a middle hydrophobic domain rich in alanine, and a C-terminal HGK-repeat domain (Figure 1a), which result in low compositional complexity in GPRPs [2,18]. Meanwhile, GPRPs have been found to comprehensively express in various tissues and developmental stages in both soybean and *Arabidopsis* (Figure 2a,b) [2], and more than 28 non-redundant proteins were found to potentially interact with AtGPRP3 (unpublished data, Hui Peng and Shaobo Li). These features indicate that GPRPs play a fundamental role in a variety of physiological activities. Consistent with this conjecture, the role of GPRPs in response to both biotic and abiotic stresses have been experimentally documented [9,11]. The involvement of GPRPs in the regulation of plant growth revealed in this study further emphasizes the importance of GPRPs in plant physiology and provides a new breakthrough point to help decipher the regulation of plant growth (Figure 3).

Previous studies revealed the minor effects of CAT1 and CAT3 on catalase activity [19,20,21]. In this study, knockout of CAT2 resulted in the retarded growth of *Arabidopsis* seedlings (Figure 5), confirming that CAT2 is the major catalase involved in plant growth [16,22]. Under normal conditions, catalases can efficiently remove excessive H_2_O_2_ as homotetramers in the peroxisomes, and indole-3-acetic acid (IAA) is able to access the cell nucleus and activate the transcription of growth-related genes [23]. However, when CAT2 is knocked out, accumulated H_2_O_2_ can inhibit the biosynthesis of IAA in the cytosol and consequently impedes the growth of transformed plants [24,25]. Catalases are synthesized in cytosol and then transported to different cell compartments [16]. With the chaperone of NCA1, catalases enter the peroxisomes, where these enzymes deplete H_2_O_2_ [16]. Both CAT2 and CAT3 interacted with AtGPRP3 in the nucleus (Figure 4b), indicating a possible reduction of catalases in the peroxisomes. The activity of catalases in the peroxisomes and the level of H_2_O_2_ were not determined in this study, but it is possible that the insufficient number of catalases in the peroxisomes leads the accumulation of H_2_O_2_ and further reduces the level of IAA. This assumption must be examined by further studies. Catalase transportation requires the participation of other components, at least in some cases. It is known that catalase transportation to peroxisomes and activity maintenance require the chaperone function of the gene *NCA1* [16]. It is unknown how catalases (CAT2 and CAT3) are transported into the nucleus. However, more GPRPs can retain more catalases in the nucleus, which results in fewer catalases in the peroxisomes, the location of catalase activation. In this way, overexpression of *AtGPRP3* can lead to growth retardation in *Arabidopsis* by inhibiting the activity of IAA.

AtGPRP3 interacts with CAT2 and CAT3 but not CAT1 (Figure 4a,b). Several proteins, such as LSD1 and NCA1, have been shown to interact with catalases and regulate their activity in response to environmental stresses [16,21]. Zinc fingers are required for the interaction of LSD1 with catalases [21]. The N-terminal RING-type zinc finger of NCA1 is also required for activating CAT2 activity, although the C-terminal tetratricopeptide repeat (TPR) helical domain alone is enough for the interactions with CAT2 [16]. BAK1 mediates light intensity to phosphorylate and activates catalases to regulate plant growth and development [20]. However, neither the zinc finger nor TPR exist in the GPRPs (Figure 1a), indicating a new type of interaction of GPRPs with catalases. GPRPs contain three conserved but function-unknown domains, an N-terminal XYPP domain, a central hydrophobic region, and a C-terminal HGK (Figure 1a). The central hydrophobic region that has the features of a transmembrane domain has been shown to interact with microsomal membranes in vitro [1] and is thus unlikely to be involved in the interactions with catalases. The N-terminal XYPP-repeat motif of annexin VIIs can interact with sorcin in a Ca^2+^-dependent manner [26]. The XYPP repeats of synaptophysins are also proposed to interact with cytoplasmic components [4]. Thus, the XYPP domain of AtGPRP3 might be involved in the interactions with catalases. On the other hand, histidine- and glycine-rich regions were found to be directly associated with protein–protein interactions in some cases [5,6], suggesting a possible involvement of HGK repeats in the interactions of AtGPRP3 with catalases. An interaction test with mutated AtGPRP3 and catalase is needed to identify the domains and critical residues responsible for this interaction. Crystal structure analysis could help further help elucidate the roles of the three domains involved in GPRP-mediated catalase activity inhibition and determine the functional complex of AtGPRP3 and a catalase.

*AtGPRP3* was globally expressed in the entire plant (Figure 2a,c), and modification of the *AtGPRP3* transcriptional level could significantly change the fresh weights of seedlings (Figure 3), suggesting the involvement of GPRPs in plant growth and development. Other studies have documented that the expression of GPRPs is induced by both biotic and abiotic stresses [2,9,10], and the exotic expression of a GPRP confers cold/drought and phytopathogen resistance to yeast and tobacco, respectively [9,11], suggesting the positive role of GPRPS in the responses against abiotic and biotic stresses. Since AtGPRP3 physically interacts with CAT2 and CAT3 (Figure 4a,b), two major catalases scavenging H_2_O_2_ in cells, it is possible that AtGPRP3 helps balance the processes of plant development and stress response by affecting the homeostasis of H_2_O_2_. To examine this possibility, future efforts should be made to investigate the physiological significance of the interactions of AtGPRP3 and catalases. Nevertheless, our results together with previous findings suggest that AtGPRP3 is a key element that regulates both plant growth and stress response, potentially through CAT2 and CAT3.

## 4. Materials and Methods

### 4.1. Plant Growth and Growth Rate Determination

*A. thaliana Col-0* was selected as the wild-type (WT) material in this study. After being sterilized and rinsed, *Arabidopsis* seeds of WT and transgenic plants were kept in the dark at 4 °C for 3 days, and then sown on a 1/2 MS medium for germination under 16 h of light and 8 h of dark at 23 °C. For growth rate determination, 4-day-old seedlings were transferred to a 1/2 MS medium at pH 5.8 or pH 8.0 and grown in an incubator (Percival, IA, USA) for 7 days under controlled conditions (16 h light/8 h dark). The fresh weights of five shoots of 11-day *Arabidopsis* seedlings were measured to determine the growth rates of the plants with six replicates.

### 4.2. Structural and Sequence Analysis of GPRPs

Identification of the homologs of GPRPs was achieved through BlastP on the NCBI genome database with the amino acid sequence of AtGPRP (AT5G45350, named “AtGPRP2” in this study) [1]. A total of 27 GPRPs from different species, including *Oryza sativa*, *Zea mays*, *Glycine max*, *Sorghum bicolor*, *Ipomoea batatas*, *Ipomoea trifida,* and *Arabidopsis thaliana*, representing both monocotyledon and dicotyledon, were recruited for the multiple sequence alignment and phylogenetic analyses (Table S3 and Supplementary Data 1). Multiple sequence alignment of the GPRP proteins was performed using ClustalW (http://www.ebi.ac.uk/clustalw/). Phylogenetic analysis was conducted by the neighbor-joining (NJ) method using MEGA (version 4.1) with 1000 bootstrap replications [27]. Clones of the full-length ORF of *AtGPRP3* were obtained by high-fidelity PCR using specific primers (Table S1) developed from the nucleotide sequence of AT4G19200 in the NCBI database. The accuracy of the coding sequence of *AtGPRP3* was verified by Sanger sequencing. The genomic structure of *AtGPRP3* was analyzed and presented by the online software GSDS (http://gsds.cbi.pku.edu.cn/index.php).

### 4.3. Plasmid Construction and Plant Transformation

*AtGPRP3* knockout mutants were generated using the CRISPR/Cas9 system following the reported method with minor modifications [28]. Two mutation targets for *AtGPRP3* were selected using CRISPR RGEN Tools following the manufacturer’s instructions (http://www.rgenome.net/cas-designer/). Spacers were cloned by ligating complementary oligos into a type II restriction site (*Bsa* I), and Gateway recombination was used to incorporate guide sgRNA into the destination vector, which contained Cas9 driven by the YAO promoter for expression. To construct the overexpression vector of *AtGPRP3*, the full-length ORF missing the stop codon was amplified and inserted into a binary vector pCXSN that carries 35S promoters. To generate the GUS reporting system driven by the promoter of *AtGPRP3* (*pAtGPRP3*), a 2116-bp upstream fragment of the start codon was amplified by PCR using specific primers (Table S1) and inserted into the binary vector pCAMBIA1301 through homologous recombination [29]. All vectors were verified by DNA sequencing and then transferred into Arabidopsis using the floral-dip method [30]. Vectors carrying the CRISPR/Cas9 system, *AtGPRP3* ORF, or GUS reporting system were transferred into *Col-0* plants to obtain the *atgprp3* mutant, *AtGPRP3* overexpressing, or promoter detection plants, respectively. The *atgprp3* mutant plants were further transformed with *AtGPRP3* overexpressing vectors to generate functional complementary lines.

### 4.4. RNA Isolation and Quantitative RT-PCR Analyses

Roots, stems, rosettes, caulines, flowers, and capsules were collected from 5-week *Col-0* plants (at least 3 week) cultured under normal growth conditions. Each type of sample was independently collected three times. The collected samples were immediately and thoroughly frozen in liquid nitrogen and stored at −80 °C for future use. Total RNAs from the different tissues were isolated using a TRIzol reagent (TransGen, Beijing, China) according to the manufacturer’s instructions. First-strand cDNA was synthesized using the FastKingRTKit (with gDNase) (TransGen). Gene-specific primers for qRT-PCR were designed with Primer5 (Table S1). Quantitative real-time PCR (qRT-PCR) was carried out on an ABI StepOne™ Real-time PCR instrument (Applied Biosystems, Carlsbad, CA, USA) using Maxima SYBR Green qPCR Master Mix (Thermo, Waltham, MA, USA) in triplicate. Each reaction (20 μL) contained 1 μL of cDNA (5 ng/μL), 0.5 μL of each primer (10 μM), 10 μL of SYBR Green qPCR Master Mix (2×), and 8 μL of nuclease-free water. The qPCR procedure was set to: 95 °C for 5 min, followed by 40 cycles of 95 °C for 15 s, 60 °C for 20 s, and 72 °C for 30 s. The specificity of the PCR amplicons was examined by the melting curve (60–95 °C) with a resolution of 0.3 °C/S. Relative expression levels were calculated via the 2^−ΔΔ*C*t^ method [31].

### 4.5. ß-Glucuronidase (GUS) Staining

GUS assays were performed according to the method described previously with minor modifications [32]. Various organs from eight individual plants carrying the *pAtGPRP3*: *GUS* fusion sequence were harvested from the growth plates and immediately submerged in the GUS staining solution after removing the attached medium. Following overnight incubation (12–16 h) at 37 °C, the organs of chlorophyll were cleared for 2 days with 70% ethanol. Digitized color images of various organs were obtained via an Olympus SZX16 Zoom Stereo Microscope (Olympus, Tokyo, Japan) equipped with an Olympus E-330 camera. The experiment was independently repeated twice.

### 4.6. Subcellular Localization Analysis

To determine the subcellular localization of AtGPRP3, the ORF of the green fluorescent protein (GFP) gene was fused to the C-terminus of coding DNA sequence (CDS) of each gene under the control of the CaMV35S promoter in the p7A-GFP vector. To localize the nucleus, the CDS of a cyan fluorescence protein (CFP) was fused to the C-terminus of the CDS of the transcription factor OsGhd7 to generate the nuclear marker *35S*: *OsGhd7-CFP*. Primers for gene cloning and plasmid construction are listed in Table S1. The constructs were cotransformed into protoplasts derived from 3-week-old WT leaves by the PEG4000-mediated method described previously [33]. The fluorescence signal was observed and captured using confocal laser microscopy (LEICA DMi8, Leica, Wetzlar, Germany). The constructs also were infiltrated into 3-week-old *N. benthamiana* (tobacco) leaves as described previously [34].

### 4.7. Yeast Two-Hybrid Assay

The interaction between AtGPRP3 and CAT2 was examined by yeast two-hybrid assays according to the protocol previously described with minor modifications [35]. The full-length CDSs of AtGPRP3 and CAT2 were cloned into pGBKT7 and pGADT7, respectively. After the cotransformation, positive transformants were confirmed by PCR, followed by growth tests on SD/-Trp/-Leu and SD/-Trp/-Ade/-His/-Leu media. An SD/-Leu/-Trp/-His/-Ade medium with X-gal was used to detect α-galactosidase activity. Yeast colonies cotransformed with BD53 and AD-T were selected as the positive control, and colonies cotransformed with pGBKT7 and pGADT7 empty vectors served as the negative control.

### 4.8. BiFC Assay

BiFC assays were performed according to the previous description with minor modifications [34,36]. The AtGPRP3 encoding sequence was inserted into pSAT1-cCFP-N to form a C-terminal in-frame fusion with cCFP, and CAT1, CAT2, or CAT3 encoding sequences were introduced into pSAT1-nVenus-N to generate a C-terminal in-frame fusion with nVenus using the primers listed in Table S1. The fluorescence emissions of GFP were observed and captured under a confocal microscope (LEICA DMi8, Leica, Germany).

### 4.9. Statistical Analysis

All experimental data were the means of at least three independent replicates, and comparisons between transgenic and WT plants were performed using a one-way ANOVA with Duncan’s multiple range test. All the statistical analyses were performed using the SPSS 12.0 software.

## Supplementary Materials

The following are available online at https://www.mdpi.com/1422-0067/21/17/6168/s1.
