# Supplementary material for: The Glycine- and Proline-Rich Protein AtGPRP3 Negatively Regulates Plant Growth in Arabidopsis
Source: Int J Mol Sci. 2020 Aug 26;21(17):6168. doi: 10.3390/ijms21176168 (PMC7504531; doi:10.3390/ijms21176168)
Supplement: Supplementary file 1 [file ijms-21-06168-s001.pdf]

Supplementary Materials

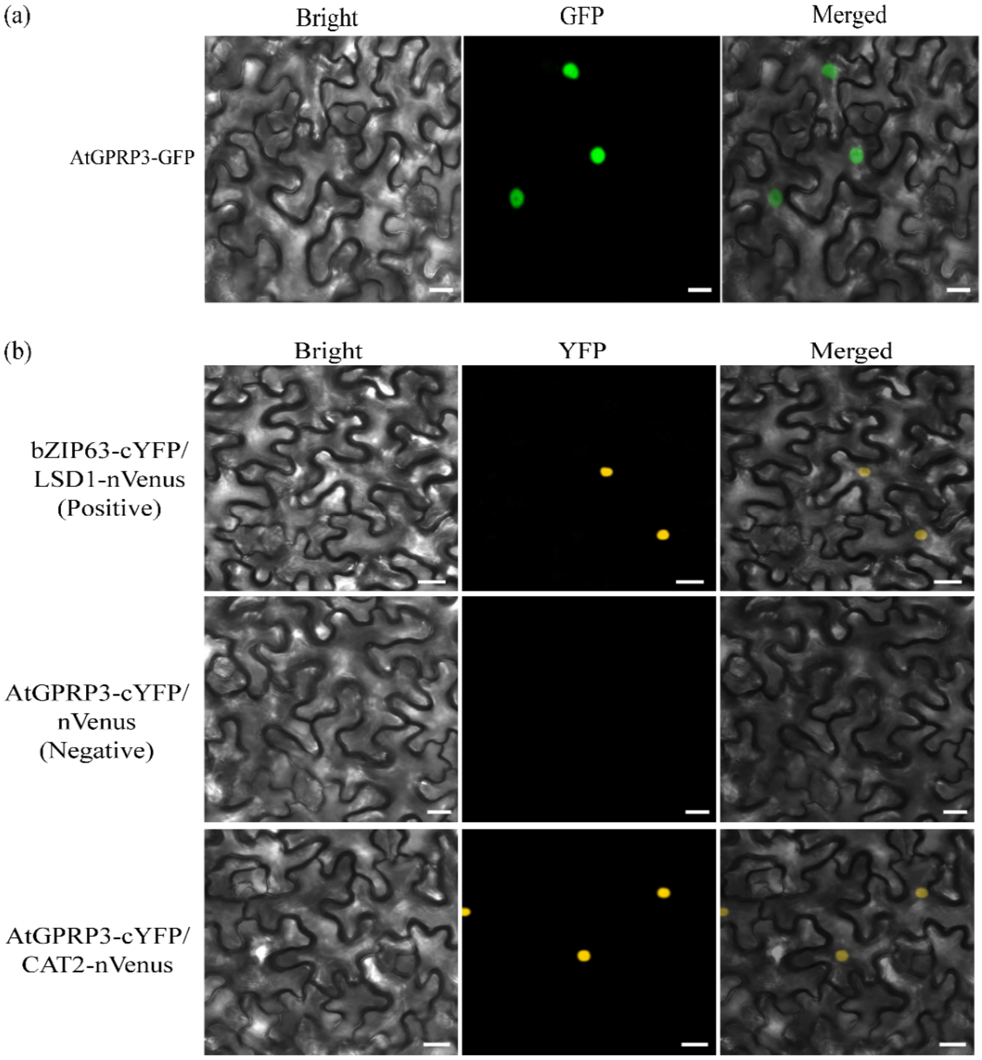

Figure S1. AtGPRP3 expressed in nucleus and interacts with CAT2. (a) AtGPRP3 expressed in nucleus. Subcellular localization of fused AtGPRP3-GFP in *Nicotiana benthamiana* epidermal cells. (b) Bimolecular fluorescence complementation (BiFC) experiment showing the interactions of AtGPRP3 and CAT2.

Table S1. Primers for functional analysis of *AtGPRP3*.

Name	Purpose	Forward primer (5′–3′)	Reverse primer (5′–3′)
SgRNA1	CRISPR_Cas9 knockout	CATTACCCACCTGCTCAAGG	CCTTGAGCAGGTGGGTAATG
SgRNA2	CRISPR_Cas9 knockout	CCACAACAAGGGTATCCTCC	GGAGGATACCCTTGTTGTGG
Cas9-P	Cas9 detection	GACAAGAAGTACAGCATCGGCCTGG	TCAGGTCCTGGTGGTGCTCGT
OXGPRP3	Overexpression	ATGGGAGGTGGTAAAGACAAG	GTCCTTATAGTCACCCATGATCTTTGTAAT CCTTCCACTTCTTGAATTTGCCTCCTC
CZ3+1301	*pGPRP3::GUS*	CTCGGTACCCGGGGATCCCAGCG AGGCTTTTGTTTGACA	CCCTCAGATCTACCATGGTGCTTTTTTA CTTTAAACCTAC
CZ3+MYC	Transgenic plant analysis	GGCGACCTCACCGAATTCCCCGGG ATGGGAGGTGGTAAAGACAAG	ATCTAGATCCGGTGGATCCTTACTTCCA CTTCTTGAATTTGC
qRTGPRP3	qRT-PCR analysis	TAGGACATGGAGGGTATGGC	TCCTCCAAACATGCCATGTT
CX-GPRP3	Transgenic plant analysis	CTGGTGTTGATTGTTGTGAATTC	GCAGGCTGATAATCCAGCAAA
YFP-GPRP3	Subcellular localization	CGCGTCGACATGGGAGGTGGTAAAGACAAG	CGCGGATCCCTTCCACTTCTTGAATT TGCCTCCTC
CFP-Ghd7	Subcellular localization	GGGGTACCATGGGGATGGCCAATGAGGA	CGGAATTCTCTGAACCATTGTCCAAGCT
Y2H-GPRP3	Yeast two-hybrid	GCTCATATGATGGGAGGTGGTAAAGACAA	AGTGAATTCTTACTTCCACTTCTTGAA TTTGC
3449-GPRP3	BiFC	CCCTCGAGCATGGGAGGTGGTAAAGACAAG	CGGAATTCCTTCCACTTCTTGAATTTGCC TCCTC
Y2H-CAT2	Yeast two-hybrid	GCTCATATGATGGATCCTTACAAGTATCGT	AGTGAATTCTTAGATGCTTGGTCTCACGT
YFP-CAT2	Subcellular localization	GCGTCGACATGGATCCTTACA AGTATCGTCCA	CGCGGATCCGATGCTTGGTCTCACGTTCAG
YFP-CAT3	Subcellular localization	ACGATACTCGAGGTCGACATGGATC CTTACAAGTATCGTCC	CACCATACTAGTGGATCCGATGCTTGGC CTCACGTTCAG
Bi-CAT1	BiFC	CCGGACTCAGATCTCGAGCATGGAT CCATACAGGGTTCGT	ACCGTCGACTGCAGAATTCGAAGTTTGGC CTCACGTTAAG
Bi-CAT2	BiFC	CCCTCGAGCATGGATCCTTACA AGTATCGTCC	CGGAATTCGATGCTTGGTCTCACGTTCAG
Bi-CAT3	BiFC	CGGACTCAGATCTCGAGCATGGAT CCTTACAAGTATCGTCC	ACCGTCGACTGCAGAATTCGATGCTT GGCCTCACGTTCAG
CAT2-SgRNA1	CRISPR_Cas9 knockout	ACTCTGGTGCTCCTGTATG	CATACAGGAGCACCAGAGT
CAT2-SgRNA2	CRISPR_Cas9 knockout	GAACGGATTCCAGAGCGTG	CACGCTCTGGAATCCGTTC
CX-KOCAT2	Transgenic plant analysis	GGAATTGCACCCAAAGTTTTGAC	ACCACGAGGGTCTCTCAAGG
qCAT2	qRT-PCR analysis	CTCCGCCTGCTGTCTGTTCT	GTGATGCGTGGGTCGGATAGG

Table S2. Detection of potential off-target sites for three different sgRNAs.

	Site	Chromosome	Position	Guide-PAM sequence	Mismatch numbers	Editing
SG1	On target (AtGPRP3)	4	10499347	ATTACCCACCTGCTCAAGGAGG	0	Yes
	Off-target 1	4	16945299	ATTAACTTCTTGCTCAAGGAGG	4	No
	Off-target 2	5	18076752	GTTTCCCACCTGATCAAAGAGG	4	No
	Off-target 3	3	2932488	TTCACCTAGCTGCTCAAAGAGG	4	No
SG2	On target (AtGPRP3)	4	10499388	GAGGATACCCTTGTTGTGGTGG	0	Yes
	Off-target 1	5	12250930	GTGGATAACCTTGTTGTGGAGG	2	No
	Off-target 2	5	16566273	GAAGGTACACTTGTTGTGGTGG	3	No
	Off-target 3	3	22271814	GAGAAAACCCTAGATGTGGTGG	4	No
SG3	On target (CAT2)	4	16702690	GAACGGATTCCAGAGCGTGTGG	0	Yes
	Off-target 1	2	14705058	GAATGGATTTCAGAGAATG TGG	4	No
	Off-target 2	3	4916841	GACTCGATTTCAGAGCATG TGG	5	No
	Off-target 3	4	17663460	GAGCGGTTACCAATGCGTG TGG	5	No
